# Silent pulmonary veins at redo ablation for atrial fibrillation: Implications and approaches

**DOI:** 10.1007/s10840-024-01750-w

**Published:** 2024-01-23

**Authors:** Peter Calvert, Wern Yew Ding, Michael Griffin, Arnaud Bisson, Ioanna Koniari, Noel Fitzpatrick, Richard Snowdon, Simon Modi, Vishal Luther, Saagar Mahida, Johan Waktare, Zoltan Borbas, Reza Ashrafi, Derick Todd, Dhiraj Gupta

**Affiliations:** 1 Liverpool Centre for Cardiovascular Science at University of Liverpool, Liverpool John Moores University and Liverpool Heart & Chest Hospital, Liverpool, UK; 2 Liverpool Heart & Chest Hospital NHS Foundation Trust, Thomas Drive, Liverpool, L14 3PE UK; 3 Centre Hospitalier Régional Universitaire et Faculté de Médecine de Tours, Tours, France; 4 Department of Cardiology, Liverpool Heart & Chest Hospital, Thomas Drive, Liverpool, L14 3PE UK

**Keywords:** Atrial fibrillation, Ablation, Redo, Reconnection, Silent pulmonary veins

## Abstract

**Background:**

Pulmonary vein isolation (PVI) is the cornerstone of atrial fibrillation (AF) ablation. Despite promising success rates, redo ablation is sometimes required. At redo, PVs may be found to be isolated (silent) or reconnected. We studied patients with silent vs reconnected PVs at redo and analysed associations with adverse outcomes.

**Methods:**

Patients undergoing redo AF ablations between 2013 and 2019 at our institution were included and stratified into silent PVs or reconnected PVs. The primary outcome was a composite of further redo ablation, non-AF ablation, atrioventricular nodal ablation, and death. Secondary outcomes included arrhythmia recurrence.

**Results:**

A total of 467 patients were included with mean 4.6 ± 1.7 years follow-up, of whom 48 (10.3%) had silent PVs. The silent PV group had had more often undergone >1 prior ablation (45.8% vs 9.8%; *p*<0.001), had more persistent AF (62.5% vs 41.1%; *p*=0.005) and had more non-PV ablation performed both at prior ablation procedures and at the analysed redo ablation. The primary outcome occurred more frequently in those with silent PVs (25% vs 13.8%; *p*=0.053). Arrhythmia recurrence was also more common in the silent PV group (66.7% vs 50.6%; *p*=0.047). After multivariable adjustment, female sex (aHR 2.35 [95% CI 2.35–3.96]; *p*=0.001) and ischaemic heart disease (aHR 3.21 [95% CI 1.56–6.62]; *p*=0.002) were independently associated with the primary outcome, and left atrial enlargement (aHR 1.58 [95% CI 1.20–2.08]; *p*=0.001) and >1 prior ablation (aHR 1.88 [95% CI 1.30–2.72]; *p*<0.001) were independently associated with arrhythmia recurrence. Whilst a finding of silent PVs was not itself significant after multivariable adjustment, this provides an easily assessable parameter at clinically indicated redo ablation which informs the clinician of the likelihood of a worse future prognosis.

**Conclusions:**

Patients with silent PVs at redo AF ablation have worse clinical outcomes.

## Introduction

Catheter ablation for atrial fibrillation (AF) is a popular approach when pursuing a rhythm control management strategy. Studies have demonstrated that ablation outperforms antiarrhythmic drugs for maintenance of sinus rhythm [1–3]. The primary benefit of AF ablation is gleaned from pulmonary vein isolation (PVI). It has long been recognised that ectopic signals arising from the pulmonary veins are responsible for triggering AF [4] and, therefore, isolating these triggers significantly reduces AF burden. It is perhaps unsurprising that patients who experience recurrence of AF post-ablation often have reconnected pulmonary veins (PVs).

In some cases, despite clinical recurrence of AF, the PVs remain isolated — so-called ‘silent’ PVs. In this situation, the optimal approach is less clear and the evidence for substrate modification and targeting of non-PV triggers is sparse [5].

We analysed cases undergoing redo AF catheter ablation at our institution and compared outcomes between those with silent vs reconnected PVs. We also sought to determine which ablation strategies were implemented and whether they affected outcomes.

## Methods

### Study design and patient selection

This study was a single-centre, retrospective observational analysis of patients who underwent redo catheter ablation procedures for AF between 2013 and 2019. Patients were identified from our institutional ablation dataset. No other inclusion criteria were applied. Patients for whom no follow-up was available were excluded. For patients with more than 2 ablations within the timeframe, we took the latest ablation to be the event of interest.

Identified records were manually reviewed to ensure accuracy of data, extract demographic and clinical data, and determine follow-up outcomes. The study was approved by our local Research & Innovation Committee.

### Outcome measures

The primary outcome was a composite of further invasive arrhythmia management (further redo ablation, non-AF ablation (e.g. atrial flutter, accessory pathway) or AV node ablation) or all-cause mortality. Secondary outcomes included the individual endpoints of the primary outcome, along with any documented atrial arrhythmia recurrence (AF, atrial flutter or atrial tachycardia).

We also performed time-to-event analysis in order to assess the association of different ablation techniques with the primary composite outcome, and with arrhythmia recurrence.

### Statistical analysis

Continuous variables were expressed as mean ± standard deviation, or median (25th quartile–75th quartile) depending upon the distribution and compared using *t*-tests or non-parametric equivalents. Statistical distribution was assessed by manual inspection of histograms and the Shapiro-Wilk test. Categorical variables were expressed as counts and percentages and compared using Fisher’s exact test. Time-to-event outcomes were analysed using Cox proportional hazard regression and Kaplan-Meier plots. Variables which with *p*<0.1 on univariable hazard regression were entered into a multivariable hazard regression model. *p*-values <0.05 were considered statistically significant. Missing data were handled by multivariable imputation by chained equations (MICE). Statistical analysis was performed in Python and R.

## Results

### Baseline characteristics

A total of 467 patients met the inclusion criteria, of whom 48 (10.3%) had silent PVs (i.e. no reconnections in any PV) at redo. Mean follow-up was 4.6 ± 1.7 years. Demographic and clinical differences between patients with silent and reconnected PVs are shown in Table 1.

**Table 1:** Demographic and Clinical Differences between patients with and without silent pulmonary veins at redo ablation

	Silent PVs (*n* = 48)	Reconnected PVs (*n* = 419)	*p*-value
Age (years, median [IQR])	61.5 [53–70]	62.0 [55–69]	0.740
Female (%)	37.5	32.0	0.516
BMI (kg/m^2^, mean ± SD)	30.2 ± 5.4	29.2 ± 5.0	0.156
Smoking status (%)			0.769
Never	66.7	70.2	
Former	29.2	26.0	
Current	4.2	3.8	
Pacemaker implant (%)	4.2	3.3	0.675
Persistent AF (%)	62.5	41.1	**0.005**
Months since prior ablation (median [IQR])	10.5 [4.0–20.3]	10.0 [5.0–20.0]	0.783
Hypertension (%)	41.7	39.1	0.757
Hypercholesterolaemia (%)	37.5	32.2	0.516
Diabetes (%)	6.2	5.5	0.742
Chronic kidney disease (%)	20.8	13.6	0.191
Ischaemic heart disease (%)	8.3	4.3	0.267
Heart failure (%)	6.2	5.3	0.734
Cerebrovascular disease (%)	10.4	8.1	0.581
CHA_2_DS_2_-VASc score (mean ± SD)	1.7 ± 1.3	1.5 ± 1.3	0.410
Significant* LA dilatation (%)	33.3	27.0	0.394
>1 prior AF ablation (%)	45.8	9.8	**<0.001**
>2 prior AF ablations (%)	14.6	1.7	**<0.001**
>3 prior AF ablations (%)	2.1	-	0.103

*AF*, atrial fibrillation; *AVNA*, atrioventricular nodal ablation; *BMI*, body mass index; *LA*, left atrial; *PV*, pulmonary vein

*Significant defined as moderate or severe based on echocardiographic parameters. Significant *p*-values are highlighted in bold

The cohorts were broadly similar, though those with silent PVs more commonly had persistent AF (62.5% vs 41.1%; *p*=0.005) and had more frequently had multiple (>1) prior AF ablations (45.8% vs 9.8%; *p*<0.001).

### Prior ablation approaches

Table 2 shows the comparison of ablation approaches undertaken in procedures *prior to* the analysed redo procedure. There were significant differences in the modality of ablation used between groups (*p* for overall effect = 0.019) — those with silent PVs had more commonly undergone radiofrequency ablation (70.8% vs 59.2%) or a combination of both radiofrequency and cryoballoon — usually over more than one procedure (8.3% vs 3.1%).

**Table 2:** Differences in prior ablation approaches between patients with silent or reconnected PVs

	Silent PVs (*n* = 48)	Reconnected PVs (*n* = 419)	*p*-value
Redo indication (%)			0.108
Atrial fibrillation	68.8	80.9	
Atypical flutter	12.5	4.8	
Typical flutter	4.2	5.3	
Unspecified NCT	-	1.0	
Research study	12.5	7.4	
Previous ablation modalities used (%)			**0.019**
Radiofrequency	70.8	59.2	
Cryoballoon	20.8	37.7	
Multiple*	8.3	3.1	
Prior PVI only (%)^†^	47.9	71.6	**0.001**
Prior CTI line (%)	33.3	22.2	0.104
Prior roof line (%)	29.2	14.1	**0.011**
Prior floor line (%)	16.7	8.1	0.061
Prior posterior wall isolation (%)^‡^	16.7	7.4	**0.047**
Prior CFAE ablation (%)	6.2	2.9	0.192
Prior mitral line (%)	20.8	6.9	**0.003**
Prior VoM ablation (%)	2.1	0	0.103
Prior SVC ablation (%)	6.2	0.2	**0.004**
Prior ablation within coronary sinus (%)	8.3	1.9	**0.026**
Prior other ablation strategy (%)^§^	12.5	1.0	**<0.001**

*Multiple refers to combination of cryoballoon and radiofrequency, either across multiple procedures (i.e. more than 1 redo) or cryoballoon PVI combined with targeted RF ablation (e.g. CTI line). ^†^Patients who had PVI only without any non-PV ablation in prior procedures. ^‡^Posterior wall isolation refers to a combination of roof and floor lines. ^§^Other strategies mainly included focal right or left atrial ablation, with small numbers of left atrial appendage isolations. Significant *p*-values are highlighted in bold

*CFAE*, complex fractionated atrial electrogram; *CTI*, cavotricuspid isthmus; *NCT*, narrow complex tachycardia; *PV*, pulmonary vein; *SVC*, superior vena cava; *VoM*, vein of Marshall

Similarly, those with silent PVs had more commonly undergone additional non-PV ablation previously, especially roof lines (29.2% vs 14.1%; *p*=0.011), mitral lines (20.8% vs 6.9%; *p*=0.003), superior vena cava (SVC) ablation (6.2% vs 0.2%; *p*=0.004), ablation within the coronary sinus (CS) (8.3% vs 1.9%; *p*=0.026), or other ablation strategies, which mainly comprised focal left and/or right atrial ablation (12.5% vs 1.0%; *p*<0.001).

As our data were collected over several years (2013–2019), operator practice and available technology advanced across this period. Generally, cryoballoon PVI was performed using standard cryoballoon techniques with the Arctic Front or Arctic Front Advance (Medtronic) catheters. PVI with radiofrequency was performed using wide area circumferential ablation, with contact force sensing technology, and guided by the prevailing metric at the time, per operator preference (e.g. force-time integral, ablation index or impedance drop).

### Ablation strategies at redo

Table 3 shows a comparison of procedural approaches between the two cohorts at the *latest* redo procedure (event of interest). Most cases in both arms had general anaesthesia and ultrasound-guided femoral venous access and were mapped with the CARTO (Biosense Webster, Irvine, CA) system.

**Table 3:** Ablation strategies utilised at redo procedure when PVs were silent vs reconnected

	Silent PVs (*n* = 48)	Reconnected PVs (*n* = 419)	*p*-value
General anaesthesia (%)	79.2	78.8	>0.999
Ultrasound-guided access	97.9	95.2	0.711
Mapping system (%)			0.750
CARTO	89.6	89.3	
Rhythmia	8.3	7.9	
Precision	-	1.4	
Acutus	-	0.5	
None	2.1	1.0	
Any ablation performed (%)	85.4	100	**<0.001**
Ablation modalities used (%)			**<0.001**
Radiofrequency	85.4	99.0	
Cryoballoon	-	1.0	
None used	14.6	-	
Ablation duration (min; median [IQR])	14.5 [9.8–21.3]	16.0 [10–23]	0.829
Pulmonary vein re-ablation*	8.3	100	**<0.001**
CTI line (%)	18.8	22.2	0.713
Roof line (%)	31.2	19.6	0.089
Floor line (%)	18.8	13.4	0.376
Posterior wall isolation (%)^†^	16.7	10.3	0.270
CFAE ablation (%)	8.3	3.3	0.102
Mitral line (%)	22.9	7.4	**0.002**
VoM ablation (%)	-	0.2	>0.999
SVC ablation (%)	20.8	6.9	**0.003**
Ablation within coronary sinus (%)	4.2	2.1	0.315
Other ablation strategy (%)^‡^	31.2	4.8	**<0.001**

*PV re-ablation refers to re-isolation in the reconnected group; in the silent PV group, this relates to further ablation around the PVs despite existing isolation, for example, making the WACA more antral. ^†^Posterior wall isolation refers to a combination of roof and floor lines. ^‡^Other strategies primarily consisted of focal right or left atrial ablation. Significant *p*-values are highlighted in bold

*CFAE*, complex fractionated atrial electrogram; *CTI*, cavotricuspid isthmus; *PV*, pulmonary vein; *SVC*, superior vena cava; *VoM*, Vein of Marshall; *WACA*, wide area circumferential ablation

Whilst all patients with reconnected PVs had further ablation performed, in 7 (14.6%) of those with silent PVs, further ablation was not performed. Five of these cases were mandated redo procedures as part of the PRESSURE clinical trial [6] — hence, for this minority group, silent PVs were a positive outcome. In the remaining two patients, the decision not to perform any further ablation was made on clinical grounds. Ablation was almost exclusively performed with radiofrequency in both groups, and ablation times were similar (median 14.5 min vs 16 min; *p*=0.829).

Pulmonary vein re-ablation was performed in all patients with reconnected PVs. A minority (8.3%) of those with silent PVs had further PV ablation, often to make the ablation circle more antral.

Non-PV ablation was more frequently delivered in the silent PV group, including mitral lines (22.9% vs 7.4%; *p*=0.002), SVC ablation (20.8% vs 6.9%; *p*=0.003) and other ablation strategies, again mostly consisting of focal left and/or right atrial ablation (31.2% vs 4.8%; *p*<0.001). Non-PV strategies were implemented at operator preference and generally were either empirical or targeted a specific substrate such as an atypical flutter circuit or non-PV trigger.

### Primary composite outcome

The primary composite outcome occurred in 12 (25.0%) patients with silent PVs vs 58 (13.8%) of those with reconnected PVs (*p*=0.053).

On univariable Cox proportional hazard regression, silent pulmonary veins were associated with an increased risk of the primary composite outcome (HR 1.95 [95% CI 1.05–3.62]; *p*=0.036); however, this became non-significant after multivariable adjustment (aHR 1.04 [95% CI 0.47–2.29]; *p*=0.919).

A similar trend was observed with several variables which were significant on univariable regression but were rendered non-significant by multivariable adjustment, as shown in Table 4. The only variables which remained significantly associated with the primary composite endpoint after adjustment were female sex (aHR 2.35 [95% CI 2.35–3.96]; *p*=0.001) and ischaemic heart disease (aHR 3.21 [95% CI 1.56–6.62]; *p*=0.002). The proportional hazards assumption was met for the global model (Schoenfeld test *p*=0.730).

**Table 4:** Cox proportional hazard regression for predictors of the primary composite outcome

Parameter	Univariable hazard ratio (95% CI)	*p*-value	Multivariable adjusted HR (95% CI)	*p*-value
Age (per year)	1.05 (1.02–1.08)	**<0.001**	1.02 (0.99–1.05)	0.147
Female sex	1.87 (1.17–2.99)	**0.009**	2.35 (1.39–3.96)	**0.001**
BMI (per unit)	0.99 (0.94–1.03)	0.536		
CHA_2_DS_2_VASC score (per unit)	1.49 (1.27–1.74)	**<0.001**	§	
Hypertension	2.14 (1.27–1.74)	**<0.001**	1.62 (0.97–2.71)	0.065
Hypercholesterolaemia	1.73 (1.08–2.77)	**0.023**	1.17 (0.70–1.98)	0.546
Diabetes	1.37 (0.55–3.42)	0.493		
Chronic kidney disease	1.62 (0.88–2.96)	0.119		
Ischaemic heart disease	3.83 (1.96–2.96)	**<0.001**	3.21 (1.56–6.62)	**0.002**
Heart failure	2.09 (0.96–4.56)	0.065	2.15 (0.94–4.92)	0.069
Cerebrovascular disease	1.37 (0.63–2.99)	0.433		
Persistent AF	1.92 (1.19–3.08)	**0.007**	1.60 (0.94–2.72)	0.081
Significant* LA dilatation	2.00 (1.24–3.24)	**0.005**	1.52 (0.91–2.52)	0.107
>1 prior AF ablation	2.21 (1.26–3.87)	**0.006**	1.41 (0.70–2.84)	0.331
Prior non-PV ablation^†^	1.88 (1.18–3.01)	**0.008**	1.33 (0.78–2.28)	0.296
Silent PV at redo	1.95 (1.05–3.62)	**0.036**	1.04 (0.47–2.29)	0.919
CTI line	1.29 (0.76–2.21)	0.351		
Roof line	1.49 (0.87–2.55)	0.146		
Floor line	0.96 (0.47–1.92)	0.897		
CFAE ablation	1.13 (0.36–3.61)	0.830		
Mitral line	2.10 (1.13–3.91)	**0.020**	1.18 (0.57–2.45)	0.664
SVC ablation	1.12 (0.48–2.59)	0.795		
Ablation within CS	0.58 (0.08–4.19)	0.591		
Other ablation^‡^	2.79 (1.46–5.32)	**0.002**	1.66 (0.76–3.63)	0.200

The primary outcome was a composite of all-cause mortality or further invasive management of AF (further redo ablation, non-AF ablation or AV node ablation). *Significant defined as moderate or severe based on echocardiographic parameters. ^†^Non-PV ablation is a composite including roof line, floor line, CFAE, mitral line, VOM, ablation within the coronary sinus, CTI line, SVC ablation or any other non-PV technique. ^‡^Other ablation techniques primarily consisted of focal left or right atrial ablation. ^§^CHADSVASC score left out of multivariable model due to multicollinearity with individual components which were included. Significant *p*-values are highlighted in bold

*AF*, atrial fibrillation; *AVNA*, atrioventricular nodal ablation; *BMI*, body mass index; *HR*, hazard ratio; *LA*, left atrial; *PV*, pulmonary vein

### Secondary outcomes

All-cause mortality was higher in the silent PV group but did not meet statistical significance (8.3% vs 3.3%; *p*=0.102). No patients in the silent PV arm underwent further AF or non-AF ablation (other than AV node ablation) within the study timeframe, while small numbers of those with reconnected PVs underwent these procedures (2.4% and 2.6% respectively). Subsequent AV node ablation was significantly more common in those with silent PVs (18.8% vs 7.2%; *p*=0.012).

Documented arrhythmia recurrence was frequent in both groups, although higher in the silent PV group (66.7% vs 50.6%; *p*=0.047). Several factors, including hypertension, persistent AF, and roof lines and mitral lines performed at redo, were associated with an increased risk of arrhythmia recurrence on univariable hazard regression (Table 5). Following multivariable adjustment, the only independent predictors of arrhythmia recurrence were moderate or severe left atrial (LA) enlargement (aHR 1.58 [95% CI 1.20–2.08]; *p*=0.001) and more than one prior AF ablation (aHR 1.88 [95% CI 1.30–2.72]; *p*<0.001). The proportional hazards assumption was met for the global model (Schoenfeld test *p*=0.803).

**Table 5:** Cox proportional hazard regression for predictors of any arrhythmia recurrence

Parameter	Univariable hazard ratio (95% CI)	*p*-value	Multivariable adjusted HR (95% CI)	*p*-value
Age (per year)	1.01 (1.00–1.02)	0.059	1.00 (0.99–1.02)	0.491
Female sex	1.16 (0.89–1.51)	0.267		
BMI (per unit)	1.02 (0.99–1.04)	0.162		
CHA_2_DS_2_VASC score (per unit)	1.11 (1.01–1.22)	**0.023**	§	
Hypertension	1.36 (1.06–1.76)	**0.016**	1.16 (0.88–1.53)	0.308
Hypercholesterolaemia	1.36 (1.06–1.68)	**0.050**	1.10 (0.83–1.46)	0.498
Diabetes	1.18 (0.69–2.02)	0.558		
Chronic kidney disease	1.03 (0.72–1.48)	0.865		
Ischaemic heart disease	1.49 (0.88–2.52)	0.135		
Heart failure	1.22 (0.71–2.09)	0.475		
Cerebrovascular disease	0.85 (0.52–1.39)	0.512		
Persistent AF	1.37 (1.07–1.76)	**0.014**	1.07 (0.81–1.41)	0.618
Significant* LA dilatation	1.73 (1.33–2.26)	**<0.001**	1.58 (1.20–2.08)	**0.001**
>1 prior AF ablation	2.14 (1.56–2.93)	**<0.001**	1.88 (1.30–2.72)	**<0.001**
Prior non-PV ablation^†^	1.35 (1.04–1.76)	**0.024**	0.95 (0.71–1.29)	0.756
Silent PV at redo	1.57 (1.08–2.28)	**0.017**	1.13 (0.76–1.68)	0.556
CTI line	0.96 (0.71–1.30)	0.800		
Roof line	1.52 (1.13–2.03)	**0.005**	1.25 (0.92–1.70)	0.152
Floor line	1.15 (0.81–1.64)	0.445		
CFAE ablation	0.72 (0.36–1.46)	0.368		
Mitral line	1.84 (1.27–2.67)	**0.001**	1.13 (0.73–1.73)	0.592
SVC ablation	1.28 (0.83–1.97)	0.263		
Ablation within CS	1.33 (0.63–2.83)	0.453		
Other ablation^‡^	1.27 (0.81–1.98)	0.301		

*Significant defined as moderate or severe based on echocardiographic parameters. ^†^Non-PV ablation is a composite including roof line, floor line, CFAE, mitral line, VOM, ablation within the coronary sinus, CTI line, SVC ablation or any other non-PV technique. ^‡^Other ablation techniques primarily consisted of focal left or right atrial ablation. ^§^CHADSVASC score left out of multivariable model due to multicollinearity with individual components which were included. Significant *p*-values are highlighted in bold

*AF*, atrial fibrillation; *AVNA*, atrioventricular nodal ablation; *BMI*, body mass index; *HR*, hazard ratio; *LA*, left atrial; *PV*, pulmonary vein

**Fig. 1:**
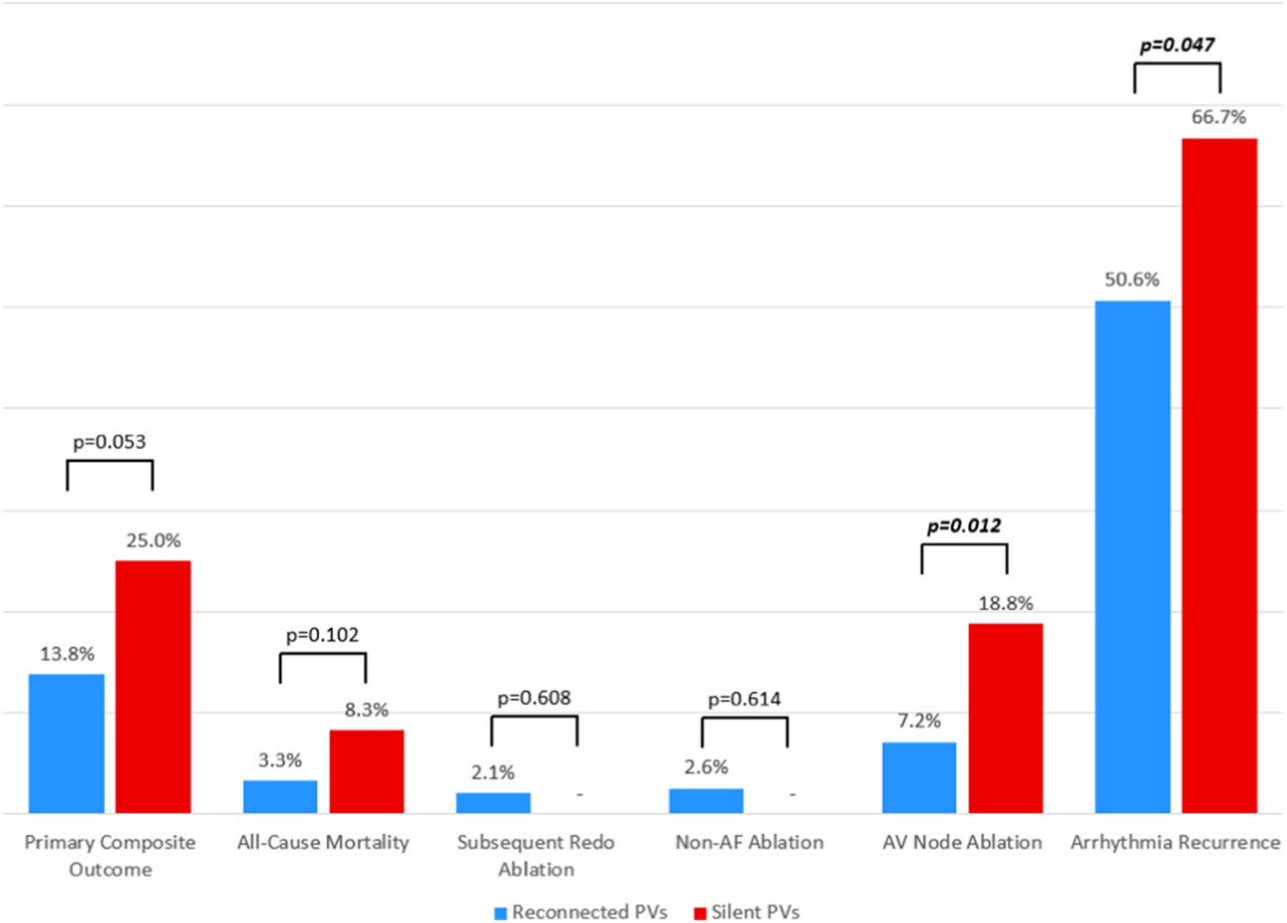
Comparison of outcomes between patients with and without silent pulmonary veins at redo ablation. The primary outcome was a composite of all-cause mortality or further invasive management of AF (further redo ablation, non-AF ablation or AV node ablation). Note that exact percentages for components of the primary composite outcome do not directly sum up as some patients had more than one outcome (e.g. AV node ablation and subsequent mortality). AF, atrial fibrillation; AV, atrioventricular

**Fig. 2:**
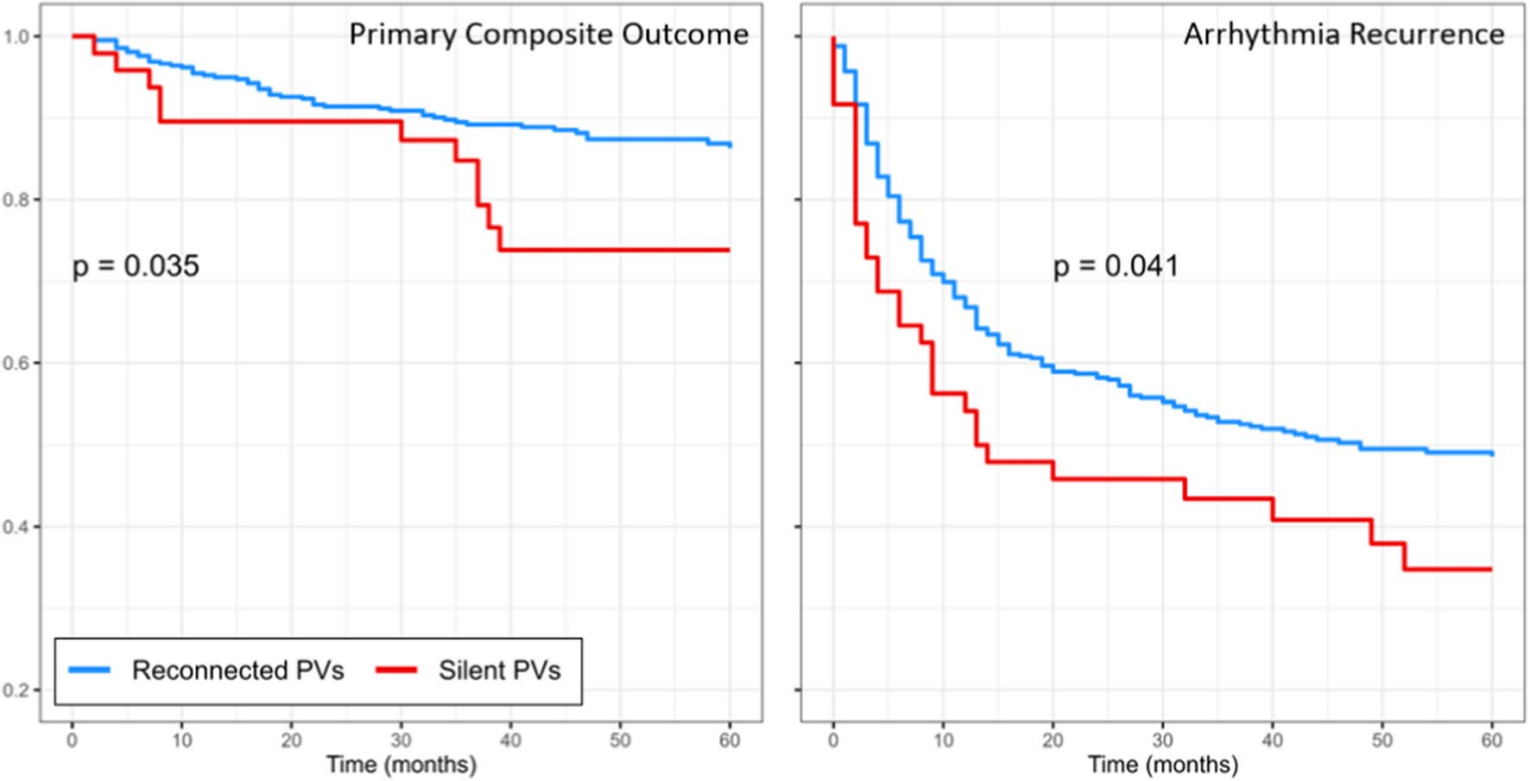
Crude Kaplan-Meier curves for the primary composite outcome (left) and any arrhythmia recurrence (right) stratified by pulmonary vein reconnection status. Note: Kaplan-Meier curves censored to 60 months as low numbers remained beyond this timepoint. Crude curves are shown as adjusted curves would be superimposed due to silent PVs being non-significant on Cox regression analysis (Tables 4 and 5)

Overall outcome comparisons are shown in Fig. 1. Kaplan-Meier curves comparing silent vs reconnected PVs with respect to the primary composite outcome, and to arrhythmia recurrence, are shown in Fig. 2.

## Discussion

In this study, our primary findings were:

1. Approximately 90% of patients undergoing redo AF ablation had reconnected PVs.
2. Patients with silent PVs had more commonly undergone >1 prior ablation, with more frequent application of non-PV ablation techniques such as mitral lines and SVC ablation.
3. Patients with silent PVs at redo AF ablation were more likely to suffer from arrhythmia recurrence and undergo subsequent AV node ablation.
4. No particular ablation technique applied at redo demonstrated an improvement in the composite primary outcome, nor in arrhythmia recurrence. Indeed, roof lines, mitral lines and focal atrial ablations were associated with worse outcomes in some cases, though this became non-significant after multivariable adjustment.
5. With multivariable adjustment, silent PVs were not independently associated with the primary composite outcome, nor the secondary outcome of arrhythmia recurrence; however, female sex and ischaemic heart disease were independently associated with the composite primary outcome, and multiple (>1) prior ablations and moderate or severe LA dilatation were independently associated with arrhythmia recurrence.

Our findings are similar to a recent study by Aguilera and colleagues [7]. In their study too, patients with silent PVs had larger left atria, had more frequent persistent AF, had more extensive non-PV ablation performed at redo and were more likely to have arrhythmia recurrence. Our study includes fewer patients, but longer follow-up with the addition of multivariable hazard regression models. Another similar study — PARTY-PVI — studied 367 patients with silent PVs undergoing various ablation strategies and found that none of these approaches improved arrhythmia recurrence [8]. Similar to our findings, left atrial dilatation was the only significant factor associated with arrhythmia recurrence (HR 1.59 [95% CI 1.13–2.23]; *p*=0.006).

Despite this, the presence of silent PVs was associated with adverse outcomes in univariable analysis, and as this is easily assessed during a redo procedure, it may provide a valuable clinical marker of increased risk. Indeed, although worse outcomes may more directly relate to other co-variables (such as those described above), these are not always as obvious in clinical practice. For example, a patient may have diagnosed ischaemic heart disease, but this could vary from mild non-obstructive disease to severe triple vessel disease and is not truly a binary variable. On the other hand, a finding of silent PVs is much more ‘binary’ in nature, is easily assessed objectively during clinically indicated redo ablation, and provides the operator with important prognostic information.

Following adjustment, we did not see a statistically significant signal of benefit for any non-PV ablation technique. In fact, many point estimates — particularly roof lines, mitral lines and focal atrial ablation — trended towards an increased risk of the primary composite outcome, and of arrhythmia recurrence. Whilst it may be tempting to assume that these approaches increased the risk of adverse outcomes, caution is advised in making this interpretation.

Firstly, as shown in Table 1, patients with silent PVs had a greater burden of persistent AF, more comorbidities and larger left atria, all of which contribute to adverse outcomes. Secondly, almost half (45.8%) of these patients had undergone >1 prior AF ablation, compared with less than 10% of those with reconnected PVs — this may mean that non-PV ablation approaches were utilised to treat iatrogenic arrhythmia related to previous ablation procedures, which had more commonly been applied in the silent PV group as shown in Table 2.

Based on our study alone, this trend is therefore not necessarily applicable to those with simple de novo PVI. However, Mol and colleagues found a similar trend in patients undergoing first-time redo AF ablation, with 12-month arrhythmia recurrence in 48.6% of those with non-PV ablation targets vs 29.3% of those with PV ablation targets (*p*=0.001), so there exists some evidence supporting worse outcomes with non-PV ablation [9]. This study was also retrospective and observational, so unmeasured confounding is likely. Previous prospective work from our centre found a similar trend, with non-significant increases in arrhythmia recurrence in those undergoing PVI + lines vs PVI alone [10].

Our findings fit with the established evidence that most non-PV ablation techniques have failed to prove significant benefit [5, 11]. Even routine posterior wall isolation, previously considered beneficial based on observation studies and expert opinion, has recently been proven ineffective in the CAPLA study [12].

This has implications for the cost-effectiveness of redo ablation procedures, especially in publicly funded healthcare systems. The incremental benefit gained from each redo procedure is likely to be less; hence, the costs involved in performing the procedure time provide comparatively less benefit. The same can be said regarding safety — although serious adverse outcomes from AF ablations are rare, the more procedures a patient has, the more likely they are to experience such complications cumulatively.

## Limitations

Our study is subject to several limitations. Firstly, our data are observational in nature, and therefore, unmeasured confounding is likely to be present. Secondly, our dataset is from a single institution based in the UK, which may not be generalisable to other institutions or countries. Thirdly, the silent PV group was relatively small which may result in underpowering for some associations.

Our study timeframe largely predates recent advances in AF ablation. Some approaches, such as vein of Marshall (VoM) ablation, hybrid/convergent ablation and epicardial ablation, may be promising [13], but large-scale outcome data are still required.

In addition, we did not have access to some variables known to be associated with risk of arrhythmia recurrence, such as time from diagnosis to ablation [14]. Similarly, improvements in technology have resulted in better durability of PVI [15]; however, this was not within the scope of our study.

We did not measure patient-reported outcomes such as quality of life in our study. The aforementioned similar study by Aguilera *et al.* reported that mean quality of life, as measured by the Atrial Fibrillation Severity Score, improved at 12-month follow-up regardless of pulmonary vein status, and did not differ by cohort [7]. It is difficult to know whether this simply represents a strong placebo effect secondary to an invasive procedure, however.

Our study findings are hypothesis generating; our study cannot determine causal links. Future prospective research may be beneficial. Nonetheless, our findings are logical and comport with well-established clinical principles and existing evidence.

## Conclusion

Patients with silent PVs at redo AF ablation have higher rates of adverse outcomes, including arrhythmia recurrence. Non-PV ablation strategies applied at redo ablation do not appear beneficial. These factors should be borne in mind when considering redo AF ablation, particularly in those with multiple prior ablations, as both safety and cost-effectiveness may be reduced by recurrent procedures.
